# Results of objective brushing data recorded from a powered toothbrush used by elderly individuals with mild cognitive impairment related to values for oral health

**DOI:** 10.1007/s00784-023-05407-2

**Published:** 2023-12-21

**Authors:** Johan Flyborg, Stefan Renvert, Peter Anderberg, Tobias Larsson, Johan Sanmartin-Berglund

**Affiliations:** 1 Department of Health, Blekinge Institute of Technology, 37179 Karlskrona, Sweden; 2 Faculty of Health Sciences, Kristianstad University, 29188 Kristianstad, Sweden; 3 School of Health Sciences, University of Skövde, 54128 Skövde, Sweden

**Keywords:** Powered toothbrush, Oral health, Mild cognitive impairment, Elderly individuals

## Abstract

**Objectives:**

The study aimed to investigate how the objective use of a powered toothbrush in frequency and duration affects plaque index, bleeding on probing, and periodontal pocket depth ≥ 4 mm in elderly individuals with MCI. A second aim was to compare the objective results with the participants’ self-estimated brush use.

**Materials and methods:**

Objective brush usage data was extracted from the participants’ powered toothbrushes and related to the oral health variables plaque index, bleeding on probing, and periodontal pocket depth ≥ 4 mm. Furthermore, the objective usage data was compared with the participants’ self-reported brush usage reported in a questionnaire at baseline and 6- and 12-month examination.

**Results:**

Out of a screened sample of 213 individuals, 170 fulfilled the 12-month visit. The principal findings are that despite the objective values registered for frequency and duration being lower than the recommended and less than the instructed, using powered toothbrushes after instruction and information led to improved values for PI, BOP, and PPD ≥ 4 mm in the group of elderly with MIC.

**Conclusions:**

Despite lower brush frequency and duration than the generally recommended, using a powered toothbrush improved oral health. The objective brush data recorded from the powered toothbrush correlates poorly with the self-estimated brush use.

**Clinical relevance:**

Using objective brush data can become one of the factors in the collaboration to preserve and improve oral health in older people with mild cognitive impairment.

**Trial registration:**

ClinicalTrials.gov Identifier: NCT05941611, retrospectively registered 11/07/2023.

## Introduction

In most parts of the world, life expectancy is increasing, and many countries have a square-formed population pyramid with growing groups of older people [1–3]. This development also increases the presence of mild cognitive impairment and various forms of dementia [4, 5]. In 2010, it was estimated that 35 million people worldwide were affected by dementia, which is expected to increase to 115 million individuals by 2050 [5].

An increasing part of older people also retain their teeth throughout life [6]. Dementia and cognitive impairment have been reported to deteriorate oral health [6]. Gingivitis and deep periodontal pockets are more common in individuals with cognitive impairment than in healthy individuals [7–9]. Lack of hygiene measures results in increased growth of the bacterial flora in the oral biofilm. As a result, homeostasis will break, leading to gingivitis and degradation of supporting periodontal tissue. Therefore, the restoration and presence of a biofilm compatible with oral health are essential. Impaired motor skills [10] is a common symptom of mild cognitive impairment (MCI) and dementia [11]. This may affect the possibility of maintaining good oral hygiene measures. Lack of proper brushing techniques and sometimes difficulty remembering to brush the teeth results in increased plaque accumulation and dysbiosis of the dental biofilm [12, 13]. This may increase caries and periodontitis, resulting in physical, mental, and financial suffering for the affected individuals and great resource demands on society and healthcare.

Finding simple and inexpensive methods to preserve and improve oral health in older people with cognitive impairment is essential to obtaining good general health and quality of life. A meta-analysis from the Cochrane Institute showed that powered toothbrushes provide a statistically significant advantage compared to manual toothbrushes in terms of reduction of plaque and gingivitis in both the short and long term. The included studies showed a high level of heterogeneity that was not explained by different brush models but was more likely due to different brush patterns, frequency, and duration [14].

Using a powered toothbrush among individuals with MCI has demonstrated that oral health can be improved [15]. The present study aimed to investigate how the true use of the powered toothbrush, in terms of frequency and duration, affects plaque index (PI), bleeding on probing (BOP), and periodontal pocket depth (PPD) ≥ 4 mm in a group of older individuals with MCI. A second aim was to compare the registered time and brush frequencies to the individual’s self-estimated usage of the powered toothbrushes.

## Methods

For this study, 213 individuals were recruited from the Swedish site of the European multicenter study Support Monitoring And Reminder Technology For Mild Dementia (SMART4MD) [16]. The Blekinge Institute of Technology carried out the Swedish site. Participants were recruited from Karlskrona municipality and the Blekinge County Council.

The inclusion criteria for this study were the same as for the Swedish site of SMART4MD [17]. Participants + 55 years of age and score 20–28 on the Mini-Mental State Examination (MMSE) [18]. Man or woman, no preference will be given to either. The participants are home care recipients.

Participants have an informal carer. Participants who take prescribed medication are responsible for their medication use.

The participants have no specific conditions that reduce their physical ability to use the application to a point that makes their participation in the project impossible, as evaluated by the responsible investigator.

The location where the participant normally resides has sufficient wireless or telephone network connectivity for them to use SMART4MD daily. For this substudy to SMART4MD, the requirement of at least ten own teeth was also added.

The present study lasted from June 2018 to October 2020 and included a screening, a baseline examination, and additional reexaminations at 6 and 12 months. All examinations were performed at a university research clinic, including oral, medical, and cognitive examinations. During the study, all participants received their usual medical and dental care.

Forty-three participants were excluded from the final analysis. Nine because they did not meet the inclusion criteria of having ten teeth, and 34 participants did not present for follow-up. All participants provided written informed consent before entering the study. During the Baseline examination, each Participant received a powered toothbrush (Oral-B Genius 9000, Proctor & Gamble, www.oralb.de) [19] and was carefully instructed on how to use the toothbrush and recommended using it for at least 2 min every morning and evening. No restrictions were imposed on the use of other oral health products.

The Oral-B application was installed on the participant’s mobile phones or computer tablets. Participants were instructed verbally and in writing on transferring data from the powered toothbrush to the application. They were encouraged to do this every fortnight as the brush can store data from a maximum of 30 brushing sessions. The application registers the number of brush sessions per day and the average brushing time per session. On ten occasions, two randomly chosen toothbrushes from the test battery were tested against an external timer. The measurements were carried out with operating sessions from 20 to 200 s. The apps’ presentations noted no significant difference between the brushes and the external timer. To investigate the function of the Bluetooth transmission, an electrical fault was simulated by the battery in one brush being disconnected during an ongoing brushing session. The app then interrupted the registration of brushing time. Registration of the brushing session takes place if the operation has been longer than 30 s.

Oral, medical, and cognitive examinations were carried out during the revisits. All brush data was photographed in the Oral-B application of the participant’s mobile phones and tablet computers. If the participant did not use the application, data was transferred from the powered toothbrush to devices in the clinic to investigate whether the brush had been used in the last month. If data was available, it was photographed for registration. At the 6-month visit, the toothbrush and operation of the application were reinformed and re-instructed, and after the 12-month visit, the collected brush data were registered.

The collected data were processed, and the formula (number of brushes X average brushing time/number of observation days) was used for the periods between baseline and 6 months (M6) and between M6 and 12 months (M12). The number of observation days was set at 180 for each period. The calculations result in a value, a factor, for each participant, reflecting their diligence in brushing their teeth and using the powered toothbrush. If the powered toothbrush is used for 2 min twice daily, it gives a factor value of 48. Similarly, a factor value of 24 corresponds to, for example, using the brush for 1 min twice a day or brushing for 2 min once a day. The sample was divided into three groups. Group 1 consisted of 25% of individuals with the lowest factor, and group 3 of 25% of individuals with the highest factor. Group 2 consisted of 50% with factors in between. Eight individuals had never used the brush and were excluded from the analysis. The participants who used the powered toothbrush but not the application had their last 30 brush sessions registered at the 6- and 12-month visits. Thirteen participants had on all occasions brushed twice or more per day and at least 2 min per occasion and were therefore interpolated to a factor value of 48.01.

### Oral examination

Baseline and 6- and 12-month examinations were performed by a dentist with more than 30 years of experience as a general practitioner and 4 years of experience in clinical research examinations, and a licensed hygienist, also with 30 years of experience in general dental care and for the past 10 years at a research clinic conducting clinical research examinations.

The investigators worked with examinator alignment and assessment by first going through selected survey methods and the criteria of the scoring scales and then continuing to jointly conduct the first eleven examinations to evaluate the examination routines and the consistency of the survey findings. As there is no golden standard to compare with in this type of examination as there is no flawless examiner, the discrepancy between the two examiners has been calculated. For the detection of 4 mm pockets, the difference amounted to 7.4%, for BOP to 11.6%, and for plaque surfaces to 0.3%. After that, regular joint reviews were conducted in doubtful cases and in randomly selected participants throughout the study.

Participant visits were booked randomly, so one researcher did not always examine the same participant.

The number of remaining teeth, PI, BOP, and sites with PPD ≥ 4 mm was recorded. PI was measured on each tooth’s four surfaces: mesial, distal, buccal, and lingual, according to O’Leary’s plaque index [20]. After using disclosing dye (Directa Rondells Red, Directa Dental AB, Upplands Väsby, Sweden), the presence or absence of plaque on the tooth surface and along the gingival margin was recorded. BOP and PPD ≥ 4 mm were measured mesiobuccal, distobuccal, mesiolingual/palatinal, and distolingual/palatinal on each tooth using a PCP-12 CE probe (Hu-Friedy Inc. Chicago, IL). For PI, BOP, and PPD ≥ 4 mm, dichotomous registration was used [21], and the results were presented as a percentage (the number of registered surfaces divided by the total number of surfaces × 100). Intra-oral lesions, if present, were diagnosed.

### Medical examination

Licensed research nurses conducted medical examinations and included a questionnaire on demographic data, living conditions, and the MMSE test described below.

### Cognitive test

The MMSE test [18] focuses on memory, learning, and orientation and has a score scale from 0 to 30 points.

### Statistical analysis

Statistical analyses are performed in STATA [22] statistics programs and SPSS 28:01 [23]. The collected data were analyzed using descriptive, comparative, and logistic regression analysis. Paired samples *t*-tests and independent samples *t*-tests were used for calculations of significance. One-way independent ANOVA and Fisher’s LSD post hoc test were used for between-group probability calculations for PI, BOP, and PPD at BL, M6, and M12. The results of BOP and PI are presented as relative frequency (%). Pocket has been reported as the number of PPD ≥ 4 mm in percent of the total number of probed sites.

### Questionnaire

At each visit, the participants answered a questionnaire in which the question about toothbrushing habits was answered. The answer options for manual and powered toothbrushes were never, once a week, daily, or several times a day. Table 3 shows the answers for manual and powered toothbrushes, respectively, for the participants who answered daily or several times a day as a percentage of the whole group.

## Results

One hundred and seventy individuals fulfilled the 12-month visit. The participants’ characteristics at baseline and M12 are presented in Table 1 and show no significant differences between the groups. At baseline, 38.9% used a manual toothbrush, and 61.1% claimed prior experience using a powered toothbrush.

**Table 1:** Study characteristics at baseline and M12

	Group related to factor value for diligence and use of the powered toothbrush	Age mean	Gender % M/F	Number of teeth	MMSE mean	Higher education	Dental crowns %	Salivation ml/min	Regular smoker%
BL	1 25% lowest factor	76.7	58/42	23.6	26.53	37.2	83.7	1.46	2.3
	2 50%inbetween factor	74.8	64/36	24.9	26.94	41.3	86.3	1.88	1.3
	3 25% highest factor	74.2	72/28	22.8	27.00	30.0	80.0	1.86	2.5
M12	1 25% lowest factor			23.3	27.02		88.4	1.56	2.3
	2 50%inbetween factor			24.6	28.27		86.3	2.2	2.5
	3 25% highest factor			22.4	27.95		80.0	2.2	2.5

The participants’ registered use of the powered toothbrush is presented (Table 2). Group 1 has a low level of use compared to group 3 regarding the number of brush sessions per day and the average brushing time per month. Group 1, however, shows a 99% increase in the number of brushing sessions during the second half of the study period, while groups 2 and 3 are increasing only by 19.5% and 6.7%, respectively. The average brushing time per month demonstrates a similar pattern, with an increase of 52% for group 1, 4.3% for group 2, and 2.9% for group 3, respectively.

**Table 2:** Registered brush data from participants’ powered toothbrushes at 6 and 12 months

Brush group	Total number of toothbrush sessions		Average brush time in minutes per month		Number of brushings per day	Average brush time in minutes per brush session
	BL-M6	M6-M12	BL-M6	M6-M12	BL-M12	BL-M12
1	9.33	18.56	0.25	0.38	0.08	0.32
2	98.30	117.49	1.33	1.44	0.60	1.38
3	210.43	224.45	2.08	2.14	1.20	2.10

The participants reported their self-estimated use of manual and powered toothbrushes in a questionnaire at each examination. The results are presented in Table 3. The use of the powered toothbrush increased from baseline to 12 months in all groups. In group 1, the use increased by 28.2%, in group 2 by 33.8%, and in group 3 by 25.0%. The use of manual toothbrushes decreased in all groups between BL and 12 M.

**Table 3:** Participants’ self-estimated daily or several times daily use of manual and powered toothbrushes in percent

		Manual toothbrush %		Powered toothbrush %	
		BL	M12	BL	M12
Brush group	1	60.5	43.6	48.7	76.9
	2	46.3	23.8	63.8	97.6
	3	50.0	7.5	70.0	95.0

The PI, BOP, and PPD ≥ 4 mm values from BL to M12 improved in the three brush groups between baseline and 12 M. The changes are statistically significant except for BOP in group 1 (Table 4). At BL, group 3 had 1% less plaque, 50% less PPD ≥ 4 mm, and 35% less BOP than group 1. At M12, the conditions were that group 3 had 20% less plaque, 54% less PPD ≥ 4 mm, and 39% less BOP than group 1.

**Table 4:** The brush groups’ change in values for PI, BOP, and PPD ≥ 4 mm from BL to M12

		PI			BOP			PPD ≥ 4 mm		
	Group 1, *N* = 40 2, *N* = 80 3, *N* = 39	1	2	3	1	2	3	1	2	3
BL	Mean %	45.55	38.59	44.58	20.03	13.96	12.98	16.12	10.18	8.07
M6	Mean %	38.48	27.24	31.18	15.10	8.59	9.17	13.63	8.86	6.03
M12	Mean %	36.59	30.98	29.57	13.82	9.66	7.13	11.65	7.13	5.40
*P* value BL-M12		0.009	< 0.001	< 0.001	0.065	0.003	< 0.001	0.050	0.016	0.054

## Discussion

In the present study, it was demonstrated that despite that the registered values for frequency and duration are lower in the data extracted from the app than the normally recommended use of a toothbrush (brush for 2 min twice a day), the use of a powered toothbrush may improve PI, BOP, and reduction of numbers of pockets PPD ≥ 4 mm in a group of older with MCI.

Furthermore, the results show that individuals in group 1 (indicating little or poor dental hygiene routines) also seize the opportunity to develop their ability to use the powered toothbrush. Toothbrushing aims to eliminate dental plaque from the tooth surfaces and the gingival margins to avoid plaque-induced diseases and maintain oral health. A general recommendation is to brush one’s teeth for 2 min in the morning and evening. Powered toothbrushes often have a timer function to facilitate brushing for at least 2 min. However, the profession has no consensus regarding the method, brushing time, or the number of brushing sessions needed [24]. An observational study showed that most users used similar intra-individual motion patterns with both manual and powered toothbrushes, and they persisted in their habitual motion patterns regardless of the toothbrush type [25]. Using the powered toothbrush with or without additional brush movements apparently does not affect the result [26].

In the present study, only 13.5% of participants regularly brushed twice daily for 2 min or more. This result coincides with another study where 91.7% of participants brushed for less than the prescribed 2 min in all sessions. The participants’ average brushing time was 89 s, and large variations between and within individuals were found [27]. In contrast, Ganns et al. [25] reported a total brushing duration for powered toothbrushing of 145 s and 135 s for manual toothbrushing. However, this study is performed in an experimental setting with healthy individuals brushing once first with a powered toothbrush directly, followed by brushing with a manual toothbrush or vice versa, making the results difficult to compare. We see that there is little difference in the oral health parameters between group 2 and group 3, with an average brushing time of 1.38 and 2.10 min, respectively, from BL to month 12, while the values are significantly lower in group 1, where the average brushing time is 0.32 min, indicating that a brush time of 1.5 min may be sufficient to obtain oral health. The strength of the present study is that the data collected regarding brush use is not based on the participant’s self-assessment. The data used were recorded in the powered toothbrush and transferred to a computer application without the possibility of influencing the result.

Another strength of the present study is the long observation time and the total sample size. A weakness is that this is not a randomized controlled study. However, since it would not have been possible to record brush data in the same way for a group using only a manual toothbrush, the value of such a comparison is assumed to be low. A further limitation is that the system used is a commercial system not designed for research purposes. The data is transferred between the brush and the app using Bluetooth technology. The Bluetooth module in the brush may break. However, this cannot be investigated. No participants reported problems connecting to their app. We have investigated whether there was any difference in presentation between two randomly chosen brushes, which was not detected. An article from 2020 describes the development of a Remote Oral Behaviors Assessment System (ROBAS). A commercial-powered toothbrush (Oral-B 7000; Procter & Gamble) with the same functions as the one we used in the present study is used and evaluated to collect data for timing, duration, and pressure applied. Bluetooth transmission is used for connection to the mCerebrum data collection app, in contrast to our study, using the Oral-B app. The participants used a stopwatch as a reference during their brushings, and compared to ROBAS, the absolute mean error was 1.8%. They reported connection errors to 6.7% and summarize that based on our study findings, ROBAS has a high criterion validity for measuring oral health behaviors. It can accurately and reliably monitor brushing behaviors in the home setting for extended periods [28]. A further limitation is the necessity of regular charging of the phone and toothbrush. As this study’s participants have MCI, it increases the risk of difficulties with the maintenance of the equipment, and it cannot be excluded that this may affect the results. Many previous studies have shown that introducing a powered toothbrush improves oral health [14, 29], and the reverse relationship that dental plaque can lead to periodontal diseases is well-known and described in several articles [30, 31].

Despite the fact that 86.5% of the participants in the present study used the powered toothbrush for less than 2 min and less than twice a day, its introduction, together with information and instructions, showed improved values for PI, BOP, and a reduction of the number of pockets ≥ 4 mm. Notable from the results is that at the baseline, the amount of plaque is similar in group 1 and group 3, while BOP and PPD are lower in group 3. An explanation could be that the registration method only registers the presence or absence of dental plaque and ignores the amount of plaque on the tooth surface [20].

As previously noted, no consensus exists on how toothbrushing is best performed. Toothbrushing duration has, however, been reported as the most critical parameter for reducing the amount of plaque [26].

The present study demonstrates the possibility that even limited use of a powered toothbrush, together with information and instruction, leads to reduced plaque among individuals with MCI. The present study’s design does not allow for evaluation of the impact of the information and instruction of the results since all participants have received the same information and instruction.”

The difference between the self-reported powered toothbrush uses and the objective user data recorded from the participants’ toothbrushes is extensive. For example, group 1, the participants with the lowest factor based on the frequency of use and brushing time, states that 76.9% use the powered toothbrush daily or several times daily after 12 months, while the data recorded from their toothbrushes shows 0.08 brushings per day with an average brushing time of 0.32 min per month. Corresponding values for group 3 are 95% self-reported use daily or several times daily at M12, while the recorded values show that the brush is used 1.2 times per day for 2.1 min. There are no similar studies to compare with. However, research in other fields states that differences between self-reported and objective data have many causes, such as personal characteristics and the survey context [32]. In this study, as mentioned above, it cannot be ruled out that the result is affected by technical misregistrations of brush data.

Objective brush data recorded from powered toothbrushes can be used as a decision basis for preventing impaired oral health, not only for individuals with MCI. Future research is needed to clarify the importance of frequency of use and the brush time compared with other factors when older individuals with cognitive impairment use a powered toothbrush.

## Conclusion

Despite lower brush frequency and duration than the generally recommended, using a powered toothbrush improved oral health.

The objective brush data recorded from the powered toothbrush correlates poorly with the self-estimated brush use.

## Data Availability

The datasets used and analyzed during the current study are available from the corresponding author upon reasonable request.

## Abbreviations

FDI: World Dental Federation
MCI: Mild cognitive impairment
PI: Incidence of dental plaque
BOP: Bleeding on probing
PPD: Periodontal pockets depth
MMSE: Mini-Mental State Examination
SPSS: Statistical Package for Social Science
WMA: World Medical Association Declaration of Helsinki
SMART4MD: Support Monitoring And Reminder Technology for Mild Dementia
